# Refractive stability of a new single-piece hydrophobic acrylic intraocular lens and corneal wound repair after implantation using a new automated intraocular lens delivery system

**DOI:** 10.1371/journal.pone.0238366

**Published:** 2020-09-02

**Authors:** Kazuno Negishi, Sachiko Masui, Hidemasa Torii, Yasuyo Nishi, Kazuo Tsubota

**Affiliations:** Department of Ophthalmology, Keio University School of Medicine, Tokyo, Japan; University of Missouri-Columbia, UNITED STATES

## Abstract

**Purpose:**

To investigate refractive stability and characterize corneal incision repair up to 3 months after implantation of a new hydrophobic acrylic intraocular lens (IOL) with hydroxyethylmethacrylate using a new automated IOL delivery system.

**Methods:**

This prospective case series included 50 eyes of 50 patients undergoing phacoemulsification and implantation of the Clareon^®^ CNA0T0 IOL using the AutonoMe^®^ automated delivery system in the Department of Ophthalmology, Keio University School of Medicine. The clinical data were collected from 46 eyes of 46 patients preoperatively and 1 day, 1 week, and 1 and 3 months postoperatively. Endothelial-side incision gaping, posterior incision retraction, and Descemet’s membrane detachment were recorded as present or absent using anterior-segment optical coherence tomography postoperatively.

**Results:**

The uncorrected distance and corrected distance visual acuities improved and stabilized 1 week postoperatively. The anterior chamber depth was stable from 1 week postoperatively. The subjective refraction was stable from 1 day postoperatively. Descemet’s membrane detachments and endothelial-side wound gaping were seen in 19 (41.3%) eyes and 34 (73.9%) eyes 1 day postoperatively and decreased gradually. Posterior incision retraction was seen in eight eyes (17.4%) on day 1 and increased to 19 eyes (41.3%) 3 months postoperatively.

**Conclusions:**

The Clareon IOL had excellent refractive stability from day 1 postoperatively. The AutonoMe automated delivery system enables safe IOL implantation through a 2.4-mm corneal incision, although the wound required longer than 1 month to heal postoperatively.

## Introduction

A new commercially available, foldable, single-piece, monofocal intraocular (IOL) (Clareon^®^ CNA0T0, Alcon Laboratories Inc., Fort Worth, TX, USA) uses a modified cross-linked optic material designed for enhanced clarity, minimal surface haze, and resistance to formation of glistenings [1].

The overall design of the Clareon IOL is based on the platform of its predecessor, the single-piece AcrySof IOL (Alcon Laboratories), and a hydrophilic copolymer, 2-hydroxyethylmethacrylate, was introduced instead of the phenylethylmethacrylate used in the AcrySof IOL. As a result, the water content of the Clareon IOL is higher than that of the AcrySof IOL (1.5% versus 0.4% at 35°C), although it is still classified as a hydrophobic IOL.

Several experimental and clinical studies have reported that the new lens had the lowest levels of surface and bulk inhomogeneities and optical characteristics including surface light scattering, glistenings, and chromatic aberration compared to the commercially available IOLs, those resulted in good clinical outcomes [1–10]. Several previous reports have evaluated the clinical results of the Clareon IOL with the AutonoMe^®^ automated delivery system [2, 11]; however, no reports have described the early postoperative refractive stability or the postoperative wound-repair process. The current study reports the refractive stability in the early postoperative period and the wound repair process after cataract surgery with the Clareon IOL and the AutonoMe automated delivery system.

## Patients and methods

The Keio University School of Medicine Ethics Committee approved the study protocol (Approval Number 20180264). Each patient provided written informed consent before entry into the study, which adhered to the tenets of the Declaration of Helsinki.

### Patients

**Fig 1:**
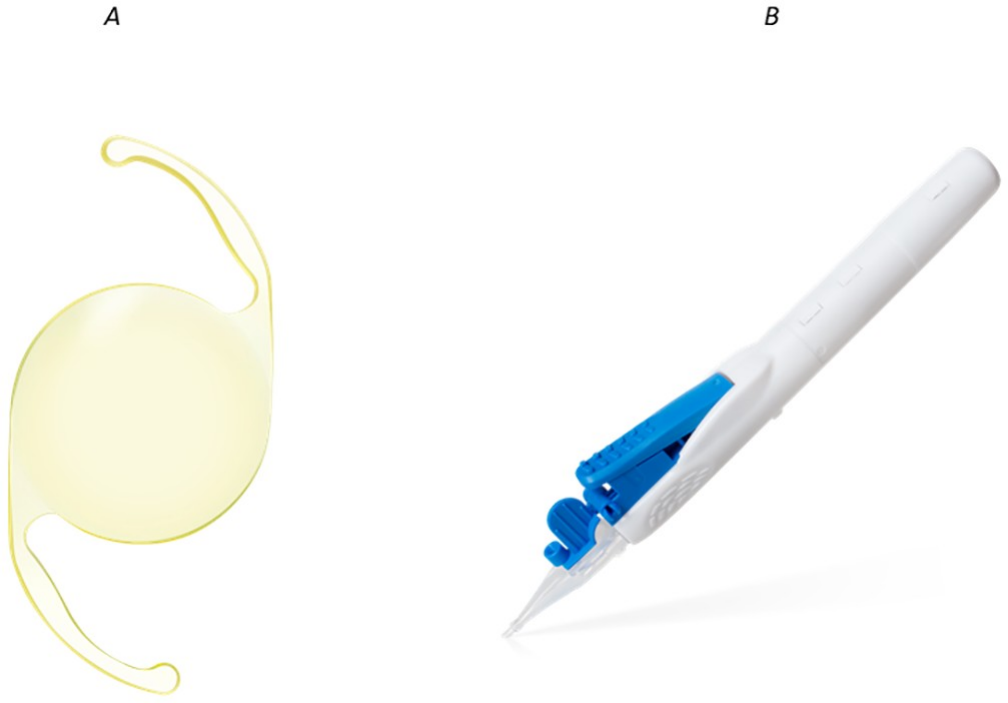
A: The Clareon CNA0T0 Intraocular Lens. B: The Autonome Automated Delivery System.

This prospective single-site observational study included 50 eyes of 50 patients who underwent cataract surgery with Clareon IOL implantation using the AutonoMe automated delivery system (Fig 1) from February to December 2019 with follow-up to 3 months postoperatively. All decisions to use the surgical intervention in this study were made as routine clinical care. The inclusion criteria were an age of 20 years and over, no ocular and systemic complications that could affect surgical outcomes, and no history of ocular surgery.

### Surgery

One experienced surgeon (K.N.) performed all surgeries using a standard technique of sutureless phacoemulsification through a 2.4-mm corneal incision using the Centurion Vision System (Alcon). An anterior capsulorhexis about 5.0 mm in diameter was created and the Clareon CNA0T0 IOL was implanted into the capsular bag using the AutonoMe automated delivery system. The cumulative dissipated energy was recorded at the end of the surgery.

### IOL

The foldable, single-piece monofocal Clareon CNA0T0 IOL was implanted in all cases. This IOL is made of a hydrophobic copolymer with a hydrophilic copolymer, 2-hydroxyethylmethacrylate, with water content of 1.5% at 35°C, a refractive index of 1.55, and a glass transition temperature of 9.1°C.

### Evaluation of clinical data

The uncorrected distance visual acuity (UDVA), corrected distance visual acuity (CDVA), subjective refraction (spherical equivalent), and subjective astigmatism were evaluated preoperatively, and at 1 day, 1 week, and 1 and 3 months postoperatively. The corneal endothelial cell density was evaluated using specular microscopy (EM-3000, Tomey Corp., Nagoya, Japan) preoperatively and 1 month postoperatively.

### Anterior-segment optical coherence tomography (AS-OCT) evaluation

AS-OCT imaging (CASIA 2, Tomey) was performed using the cornea and angle analysis mode. The anterior chamber depth (ACD) was defined as the distance from the corneal endothelial surface to the crystalline lens or the IOL parallel to the fixation axis. The AS-OCT images of the main incision were acquired on 1 day, 1 week, and 1 and 3 months postoperatively. The time-wise changes in the corneal incision site were evaluated using AS-OCT, according to a previous report [12]. The gaping of the epithelial-side and endothelial-side incisions, posterior incision retraction, and Descemet’s membrane detachment were recorded as present or absent.

The presence of recession of the edge of the posterior wound surface was considered as posterior incision retraction.

### Statistical analysis

The values are expressed as the means ± standard deviations unless otherwise specified. The data obtained in this study were analyzed using the SPSS 24 statistical software package (IBM Corp., Armonk, NY, USA). The study included four time-factor levels (1 day, 1 week, and 1 and 3 months postoperatively) at which the UDVA, CDVA, and ACD were measured. The Kolmogorov–Smirnov test was used to verify the distribution for normality. The Wilcoxon signed-rank test with Bonferroni correction was used to analyze the UDVA and CDVA. The paired t-test with Bonferroni correction was used to analyze the ACD. McNemar test with Bonferroni correction was used to analyze endothelial-side wound gaping, Descemet’s membrane detachment, and posterior incision retraction. The level of significance was set at P < 0.05.

## Results

### Patients

Fifty patients (50 eyes) were included in this study. Phacoemulsification with IOL implantation was performed successfully in all patients without any intraoperative complications. Four eyes were excluded from the analysis; three patients were lost to follow-up, and one patient developed postoperative lens-induced uveitis due to the nuclear remnant. Ultimately, data from 46 eyes of 46 patients were evaluated. The patient demographic and preoperative data are shown in Table 1. The nuclear sclerosis based on the Emery-Little classification was 1.80 ± 0.89 and the cumulative dissipated energy (%-sec) was 6.10 ± 2.61. The preoperative and postoperative corneal endothelial cell densities (cells/mm^2^) were 2,767 ± 224 and 2,601 ± 346, respectively.

**Table 1:** Patient profiles (n = 46).

Parameter	Mean ± SD
**Eyes (right/left) (n)**	23/23
**Age (years)**	72.0 ± 6.8
**Female sex, n (%)**	24 (52%)
**Emery-Little classification**	1.80 ± 0.89
**CDE**	6.10 ± 2.61
**UDVA (logMAR)**	0.92 ± 0.45
**CDVA (logMAR)**	0.23 ± 0.20
**Subjective refraction (spherical equivalent) (D)**	-4.39 ± 4.93
**Subjective astigmatism (D)**	-0.97 ± 0.20
**Anterior chamber depth (mm)**	2.83 ± 0.49
**Central corneal thickness (μm)**	535.05 ± 31.54
**Postoperative target refraction (D)**	-1.41 ± 1.25
**Corneal endothelial cell density (cells/mm**^**2**^**)**	2,767 ± 224

N = number; SD = standard deviation; CDE = cumulative dissipated energy; UDVA = uncorrected distance visual acuity; CDVA = corrected distance visual acuity; D = diopters; logMAR = logarithm of the minimum angle of resolution.

### Visual and refractive stability

**Fig 2:**
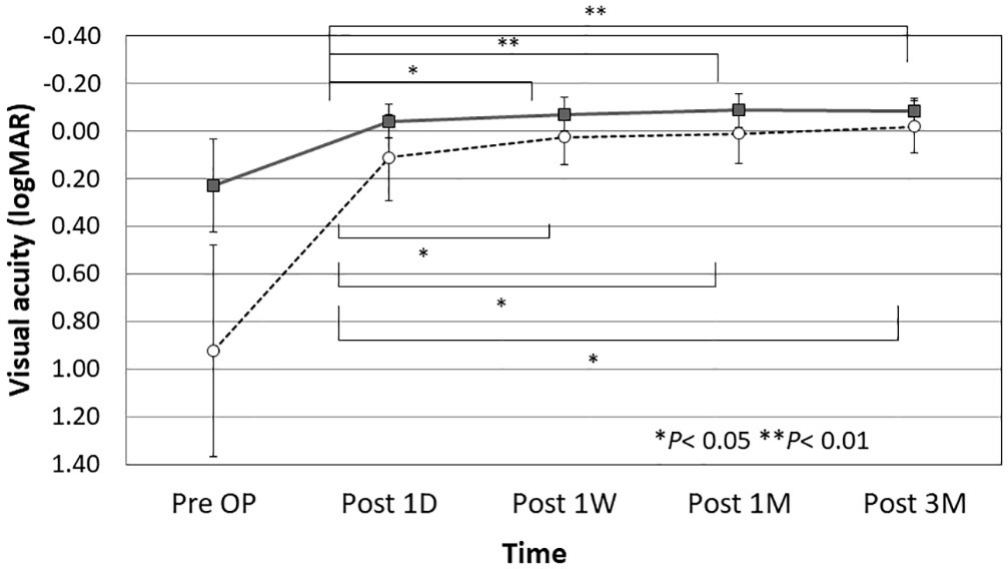
Changes in the logarithmic minimum angle of resolution (logMAR) for uncorrected distance visual acuity (UDVA) in eyes targeted for emmetropia (n = 19) and the corrected distance visual acuity (CDVA) ± with the standard deviation bar. There are no significant changes in the UDVA or CDVA after 1 week postoperatively. Pre OP = preoperative; D = days; M = months; W = weeks; circles = UCVA; squares = CDVA.

The postoperative UCVAs in 19 eyes with a postoperative target refraction of emmetropia at 1 day, 1 week, and 1 and 3 months postoperatively were 0.11 ± 0.18, 0.03 ± 0.12, 0.01 ± 0.13, and -0.02 ± 0.11, respectively. The postoperative CDVA at 1 day, 1 week, and 1 and 3 months postoperatively were -0.04 ± 0.07, -0.07 ± 0.07, -0.09 ± 0.07, and -0.09 ± 0.05, respectively, in all eyes. The UDVA and CDVA improved 1 day postoperatively, increased at 1 week postoperatively, and stabilized thereafter (Fig 2). The spherical equivalents of the subjective refraction (diopters [D]) at 1 day, 1 week, and 1 and 3 months postoperatively were -0.98 ± 1.44, -1.13 ± 1.30, -1.13 ± 1.37, and -1.11 ± 1.30, respectively, in all eyes. The subjective astigmatism (D) at 1 day, 1 week, and 1 and 3 months postoperatively were -0.73 ± 0.61, -0.70 ± 0.42, -0.66 ± 0.38, and -0.65 ± 0.42, respectively, in all eyes. The subjective refraction and subjective astigmatism changed 1 day postoperatively and stabilized thereafter.

### AS-OCT evaluation

**Fig 3:**
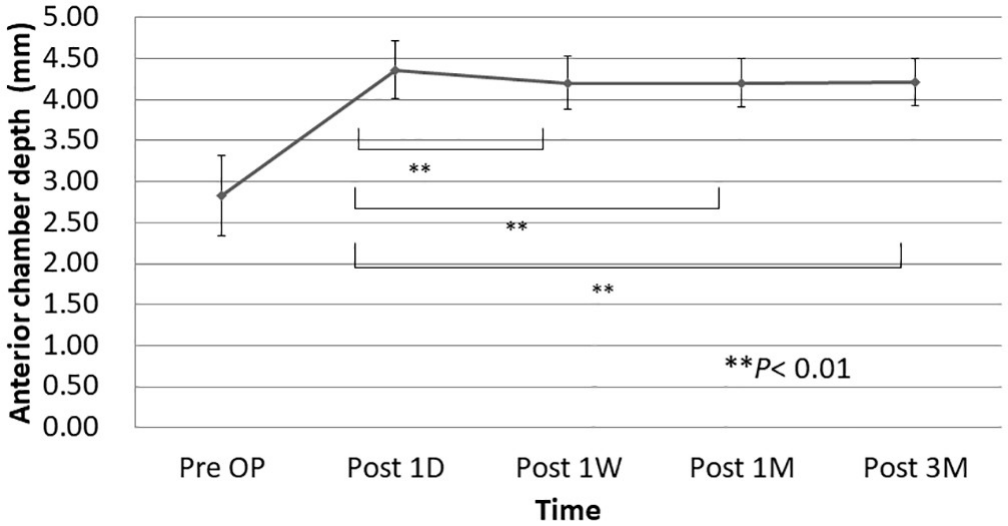
Changes in the anterior chamber depth (ACD) with the standard deviation bar. The ACD stabilized after 1 week postoperatively. Pre OP = preoperative; D = days; M = months; W = weeks. **P< 0.01.

**Fig 4:**
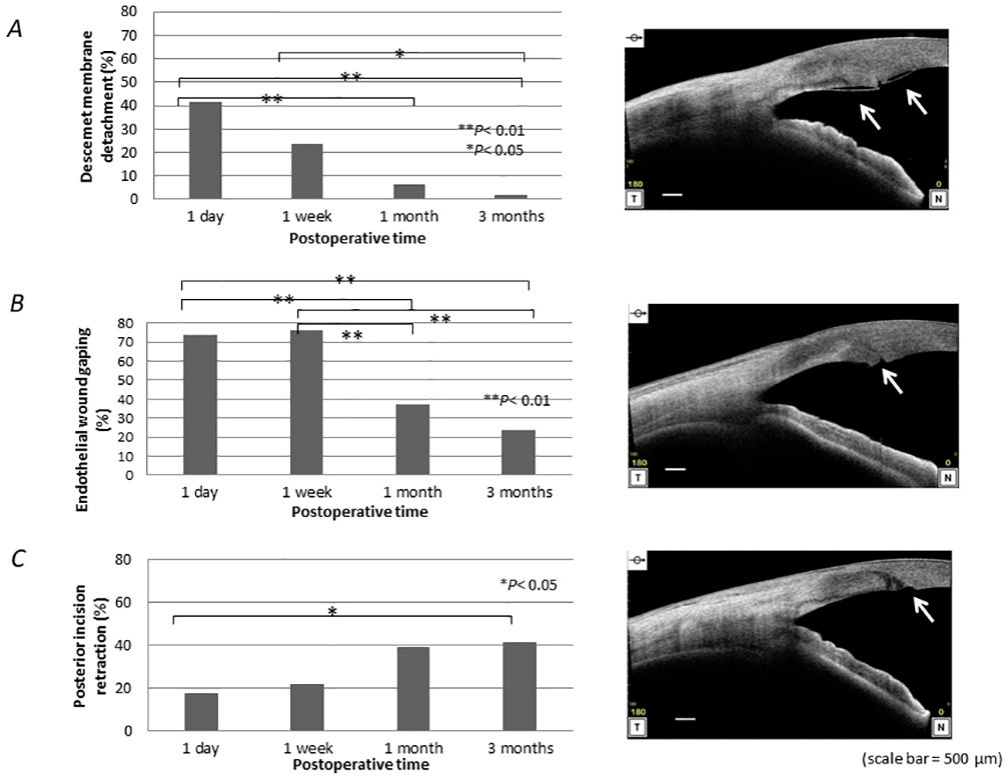
Anterior-segment optical coherence tomography evaluation. A: Descemet’s membrane detachment (arrows). B: Epithelial-side wound gaping (arrow). C: Posterior incision retraction (arrow). The left graphs show the rates of the findings. Representative photographs of the findings are shown on the right side. T = temporal; N = nasal.

The changes in the ACD are shown in Fig 3. The ACD increased at 1 day, slightly decreased at 1 week postoperatively, and stabilized thereafter. Observations of the corneal incision site evaluated by AS-OCT are shown in Fig 4. Descemet’s membrane detachments were observed in 19 (41.3%) eyes on 1 day postoperatively and decreased up to 3 months postoperatively. No epithelial-side wound gaping was seen postoperatively at any time point in any eyes. Endothelial-side wound gaping was present in 34 (73.9%) eyes on 1 day postoperatively, and the reduction continued to progress up to 1 month postoperatively. Posterior incisional retraction was observed in eight (17.4%) eyes on day 1 and increased to 19 (41.3%) eyes at 3 months postoperatively.

## Discussion

This study showed that the Clareon IOL provided good postoperative visual and refractive outcomes, both of which were very stable from day 1 postoperatively. The AutonoMe automated delivery system enabled safe IOL implantation through a 2.4-mm temporal clear corneal incision, although the wound required more than 1 month to heal.

The stability of the implanted IOL affects the postoperative refraction, optical performance, and development of posterior capsular opacification [13, 14]. The axial movement of an IOL is associated directly with the postoperative refractive stability. Ning et al. reported that the postoperative change in the ACD was correlated negatively with the postoperative refractive errors [15]. Previous studies that compared the stability of the postoperative axial movement between single-piece and three-piece IOLs [16, 17] confirmed that the refraction and ACD in eyes with single-piece IOLs were very stable compared with those in eyes with three-piece IOLs [16, 18].

In an experimental study, the Clareon CNA0T0 had the lowest levels of axial displacement and corresponding simulated dioptric power shift over all tested compression diameters compared with other one-piece acrylic IOLs, i.e., the MX60 (Bausch & Lomb, Rochester, NY, USA), ZCB00 (Abbott Medical Optics, Santa Ana, CA, USA), and XY1 (HOYA, Tokyo, Japan) IOLs [5]. The current results showed that the ACD was stable at 1 week postoperatively, and the subjective refraction stabilized on day 1 postoperatively. The results confirmed the excellent refractive stability of the Clareon IOL in clinical cases, which also can facilitate earlier spectacle prescription and more rapid visual/social rehabilitation of patients after cataract surgery. The discrepancy between the times of stabilization of the refraction and the ACD might be due to changes in the central corneal thickness postoperatively.

Regarding the postoperative wound repair, Li et al. reported a detailed comparison of the wound repair and remodeling between different incision sizes (2.2 mm versus 2.85 mm) after cataract surgery using AS-OCT [12]. According to that study, 1 day postoperatively, Descemet’s membrane detachments, which were most likely to have resulted from mechanical injury intraoperatively [19, 20], occurred in 62% and 40% of patients in the 2.20-mm and the 2.85-mm incision groups, respectively, i.e., over the calculated circumference and under the calculated circumference of 5.21 mm, respectively. This showed that the incision size below the circumference of the cartridge tip resulted in more traumatic changes in the incision.

The cartridge used for the Clareon IOL is compatible with a 2.40-mm clear corneal incision, because the recommended incision size is 2.2 mm and over according to the manufacturer. Several factors affect stretching of the incision, such as the design of the cartridge tip and insertion methods [21]. The circumference of the Clareon IOL cartridge tip was not disclosed; however, the size of the cartridge tip is the same as that of the Ultrasert delivery system (Alcon), according to the manufacturer.

Nanavaty and Kubrak-Kisza reported that the maximal post-IOL implantation percentages of stretching of the external and internal wounds using the Ultrasert delivery system were over 15% and 11% for a 2.2-mm limbal incision in porcine eyes [21]. They measured the outer size of the ellipsoid tip of the Ultrasert and reported that the long and short diameters of the tip were 2.011 mm and 1.467 mm, respectively [21]. According to those data, the calculated circumference of the ellipsoid cartridge tip of the AutonoMe is 5.497 mm, which is much larger than the circumference of a fully dilated 2.4-mm incision (4.8 mm). The current results verified that the AutonoMe can deliver an IOL with less trauma compared with the MX60 injector system [12]. Considering this, some degree of incisional stretching and trauma may occur intraoperatively using this system. However, the current results showed that the rate of Descemet’s membrane detachments was less and decreased with time and disappeared in most cases. This damage was localized to the wound, and it seemed to be repaired by endothelial cell migration and deposition of a new basement membrane [22].

Importantly, the current results showed that there were no significant differences in the UCVA and CDVA after 1 week postoperatively, which indicated that the damage was not relevant to the clinical outcome. However, the damage should be reduced in the future with improvements in the devices and techniques to increase safety and earlier recovery.

The endothelial-side wound gaping is thought to reflect wound distortion and incision trauma [23]. Li et. al reported that endothelial-side wound gaping occurred in 66% of cases 1 day postoperatively in patients with a 2.2-mm incision, i.e., which exceeded the calculated circumference, and decreased to 5.8% 3 months postoperatively. In our study, the rate of endothelial-side wound gaping was higher than that reported by Li et al. [12], and the rate of the endothelial-side wound gaping remained 24% even 3 months postoperatively. This contradicts the excellent data obtained from Descemet’s membrane detachments and warrants further investigation.

Based on previous reports, the posterior incisional retraction increased over time independent of incision size, presence of a Descemet’s membrane detachment, and wound gaping. This retraction may be associated with incisional remodeling [12] that continues slowly for a long time postoperatively [22]. The current result that posterior incision retraction increased at 3 months postoperatively was consistent with previous reports [12], and might be caused by IOL implantation when an oversized cartridge tip is used for the corneal incision width. Longer observation is necessary to investigate the relationship between posterior incisional retraction and incisional remodeling.

This study had some limitations, in that it was a single-center, prospective, observational study with a small number of cases. However, these limitations also had some merits because of the consistency of the data and differences that can be ascribed only to differences in the patients. The data obtained from this study provided new information about the refractive stability of the Clareon IOL in the early postoperative stage and safety after cataract surgery using the new AutonoMe automated delivery system.

## Supporting information

S1 Data Final data. (XLSX)
